# Suppression of Cpn10 Increases Mitochondrial Fission and Dysfunction in Neuroblastoma Cells

**DOI:** 10.1371/journal.pone.0112130

**Published:** 2014-11-12

**Authors:** So Jung Park, Doo Sin Jo, Ji Hyun Shin, Eun Sung Kim, Yoon Kyung Jo, Eun Sun Choi, Hae Mi Seo, Sung Hyun Kim, Jung Jin Hwang, Dong-Gyu Jo, Jae-Young Koh, Dong-Hyung Cho

**Affiliations:** 1 Department of East-West Medical Science, Graduate School of East-West Medical Science, Kyung Hee University, Yongin, South Korea; 2 Department of Genetic Engineering, Kyung Hee University, Yongin, South Korea; 3 School of Medicine, Kyung Hee University, Seoul, South Korea; 4 Asan Institute for Life Science, Institute for Innovative Cancer Research, University of Ulsan College of Medicine, Asan Medical Center, Seoul, South Korea; 5 School of Pharmacy, Sungkyunkwan University, Suwon, South Korea; 6 Department of Neurology, University of Ulsan College of Medicine, Asan Medical Center, Seoul, South Korea; National University of Singapore, Singapore

## Abstract

To date, several regulatory proteins involved in mitochondrial dynamics have been identified. However, the precise mechanism coordinating these complex processes remains unclear. Mitochondrial chaperones regulate mitochondrial function and structure. Chaperonin 10 (Cpn10) interacts with heat shock protein 60 (HSP60) and functions as a co-chaperone. In this study, we found that down-regulation of Cpn10 highly promoted mitochondrial fragmentation in SK-N-MC and SH-SY5Y neuroblastoma cells. Both genetic and chemical inhibition of Drp1 suppressed the mitochondrial fragmentation induced by Cpn10 reduction. Reactive oxygen species (ROS) generation in 3-NP-treated cells was markedly enhanced by Cpn10 knock down. Depletion of Cpn10 synergistically increased cell death in response to 3-NP treatment. Furthermore, inhibition of Drp1 recovered Cpn10-mediated mitochondrial dysfunction in 3-NP-treated cells. Moreover, an ROS scavenger suppressed cell death mediated by Cpn10 knockdown in 3-NP-treated cells. Taken together, these results showed that down-regulation of Cpn10 increased mitochondrial fragmentation and potentiated 3-NP-mediated mitochondrial dysfunction in neuroblastoma cells.

## Introduction

Mitochondria are constantly undergoing division and fusion in normal cells. The balance of mitochondrial dynamics influences mitochondrial morphology, distribution and function [1]. Several GTPase proteins including dynamin-related protein1 (Drp1), optic dominant atrophy 1 (Opa1), and mitofusin 1/−2 (Mfn1/2) have been identified as regulators of mitochondrial dynamics [2]–[5]. Mfn1/2 are involved in outer mitochondrial membrane (OMM) fusion with adjacent mitochondria [6]. Opa1 is responsible for inner mitochondrial membrane fusion, and maintains both mitochondrial DNA and cristae morphogenesis [7]. In contrast, mitochondrial fission relies on the Drp1 protein. Drp1 shares common mechanism with its homolog, dynamin GTPase, which is involved in endocytosis [8]. Upon stimulation of mitochondrial fission, Drp1 is translocated from the cytosol into the OMM and interacts with its receptors such as mitochondrial fission 1 (Fis1) and mitochondrial fission factor (MFF). This binding initiates the mitochondrial fission process [9]. Mitochondrial fusion functions as a cell protection mechanism, whereas massive mitochondrial fission potentiates cell death [10]. Owing to their high energy demands, mitochondrial function is particularly important in neuronal cells. Disruption of mitochondria dynamics can affect a wide range of neuronal activities such as synaptic transmission, axonal/dendritic transport, and neuronal calcium homeostasis [11]. Therefore, an imbalance in mitochondrial dynamics can contribute to neurodegenerative diseases including Huntington's disease (HD), Alzheimer's disease (AD) and Parkinson's disease (PD) [12]. HD, an autosomal dominant disorder is a genetic disease caused by mutation in the Huntingtin (*Htt*) gene. Mutant *Htt* leads to excessive mitochondrial fission and neuronal dysfunction [13]. 3-nitropropionic acid (3-NP), a mitochondrial oxidative phosphorylation complex II inhibitor, triggers a movement disorder similar in many respects to HD. 3-NP induces abnormal mitochondrial fission, prolonged energy impairments and subsequent neuronal injury [14].

Many chaperone proteins are involved in the maintenance of protein quality and function. Thus, mitochondrial chaperones regulate mitochondrial function and structure [15]. It has been reported that overexpression of DnaJA3/mtHSP40, a mitochondrial chaperone induces mitochondrial fission [16]. Depletion of another mitochondrial chaperon, prohibitin-2 leads to the loss of long Opa1 isoforms, and controls cell death [17]. In addition, we have previously shown that inhibition of mitochondrial heat shock protein mortalin/mtHSP70 promotes mitochondrial fragmentation and dysfunction in neuronal cells [18]. Chaperonin 10 (Cpn10)/heat shock 10 kDa protein 1(HSPE1) interacts with heat shock protein 60 (HSP60), and functions as a co-chaperone. The Cpn10-HSP60 complex regulates the folding of proteins imported into mitochondria [19]. Therefore, Cpn10 is important for the maintenance of normal mitochondrial structure and activity [20], [21]. Cpn10 is a multifunctional protein. The overexpression of Cpn10 has been reported alongside various tumors such as in prostate and lymphomas [22], [23]. Overexpression of Cpn10 modulates also apoptosis by increasing anti-apoptotic Bcl-2 proteins and decreasing the pro-apoptotic Bax protein [24]. In addition, Cpn10 is involved in the Ras GTPase pathway, bone marrow cell differentiation, and the IGF-1R signaling pathway [25], [26]. However, the underlying mechanism for Cpn10 regulation in mitochondria-mediated neuro-toxicity remains unclear.

In this study, we found that down-regulation of Cpn10 greatly increased mitochondrial fragmentation, and potentiated 3-NP-mediated mitochondrial dysfunction in neuroblastoma cells.

## Materials and Methods

### Cell culture and stable transfection

SK-N-MC and SH-SY5Y neuroblastoma cells were obtained from the American Type Culture Collection (ATCC). Wild type mouse embryo fibroblast (MEF) and Drp1 deficient MEF cells were generally provided by Dr. Katsuyoshi Mihara (Kyushu University, Japan) [27]. All cells were cultured at 37°C in a 5% CO_2_ incubator and maintained in Dulbecco's modified Eagle's medium (DMEM) containing 1% penicillin/streptomycin as well as 10% fetal bovine serum (Invitrogen, Carlsbad, CA). To generate stable cell line (SK/mito-YFP), SK-N-MC cells were transfected with pmito-YFP using Lipofectamine 2000 according to manufacturer's protocol (Invitrogen, Carlsbad, CA). The cells were selected by growth in selection medium containing Geneticin (1 mg/ml) for 10 days. After single cell dropping, the stable clone was selected under a fluorescence microscope.

### Reagents

The YFP-fused mito-tracker plasmid (pmito-YFP) was previously described [28]. 3-nitropropionic acid (3-NP) and N-acetylcysteine (NAC) were purchased from Sigma (St. Louis, MO USA). Mdivi-1 (3-(2,4-Dichloro-5-methoxyphenyl)-2,3-dihydro-2-thioxo-4(1H)-quinazolinone) was purchased from Enzo life sciences (Farmingdale, NY, USA). A mitoTracker probe was purchased from Invitrogen (Carlsbad, CA). The validated siRNA targeting for Cpn10 (#1, 5′-CAAAGUAGUUCUAGAUGAC-3′), (#2, 5′-GCGUGAAAGUUGGAGAUAA-3′) negative scrambled siRNA (5′-CCUACGCCACCAAUUUCGU-3′) were purchased form Dharmacon (Thermo Scientific). And previously validated Drp1 siRNA (5′-GAGGUUAUUGAACGACUCA-3′) and Opa1 siRNA (5′-CUGGAAAGACUAGUGUGUU-3′) were synthesized from Bioneer (Daejeon, Korea).

### Western blotting

For Western blotting, all lysates were prepared with protein sample buffer (62.5 mM Tris-HCl, pH 6.8, 25% glycerol, 2% SDS, 5% β-mercaptoethanol, 0.01% Bromophenol blue) (BioRad, Hercules, CA). Then the samples were separated by SDS-PAGE and transferred to PVDF membrane (BioRad). After blocking with 4% skim milk in TBST (25 mM Tris, 3 mM 140 mM NaCl, 0.05% Tween-20), the membranes were incubated over-night with specific primary antibodies at 4°C. Anti-Drp1 antibody was from BD (San Jose, CA); anti-Cpn10 antibody was from BD (San Jose, CA); anti-Actin antibody was from Millipore (Temecula, CA). For protein detection, the membranes were incubated with HRP-conjugated secondary antibodies (Pierce, Rockford, IL).

### ROS measurement

Intracellular ROS levels were assayed using a fluorescent dye, 2′,7′-dichlorofluorescein diacetate (DCFH-DA) (Invitrogen, Carlsbad, CA), which is converted to the highly fluorescent 2′,7′-dichlorofluorescein (DCF) in the presence of oxidant. Briefly, cells plated in 96-well plate were transfected with siRNA. With or without further treatment of 3-NP for 8 hr, and the cells were incubated with DCFH-DA (20 uM) in serum free medium for 30 min (excitation/emission wave length 358/485) (Victor X3, Perkinelmer). Relative ROS level was presented as the change in fluorescence of drug treated sample compared with that of control sample.

### Measurement of cellular total ATP level

SK-N-MC cells were transfected with Cpn10 siRNA for 5 days. The cells were further treated with or without 3-NP for 8 hr, and then cellular total ATP level was detected with an ATP bioluminescence detection kit according to the manufacturer's protocol (Promega, Madison, WI).

### Measurement of mitochondria membrane potential

Mitochondrial membrane potential was measured with a unique fluorescent cationic dye, JC-1 (5,5′,6,6′-tetrachloro-1,1′,3,3′-tetraethylbenzimidazolylcarbocyanine iodide, BD, San Jose, CA) that detects loss of signal of mitochondrial membrane potential. The fluorescence intensity was monitored using plate reader (PerkinElmer, Waltham, MA) at excitation and emission wavelength of 485 nm and 535 nm for monomeric form as well as 535 nm and 590 nm for JC1-aggregates form

### Apoptotic cell death analysis

Apoptotic cell death was determined by using an Annexin V-FITC/PI Apoptosis Detection Kit according to the manufacturer's protocol (BD Pharmingen, San Diego, CA). Briefly, cells transfected with Cpn10 siRNA was exposed to 3-NP for 24 hr. Then, the cells were stained with Annexin V-FITC and propidium iodide (BD Pharmingen, San Jose, CA). After staining, cell death was analyzed by using flow cytometer (BD, San Jose, CA).

### Statistical analysis

Data were obtained from least three independent experiments, and presented as means ± S.E.M. Statistical evaluation of the results was performed with one-way ANOVA. Data were considered significant at a value of *p<0.02.

## Results

### Down-regulation of Cpn10 induces mitochondrial fragmentation in neuroblastoma cells

**Figure 1:**
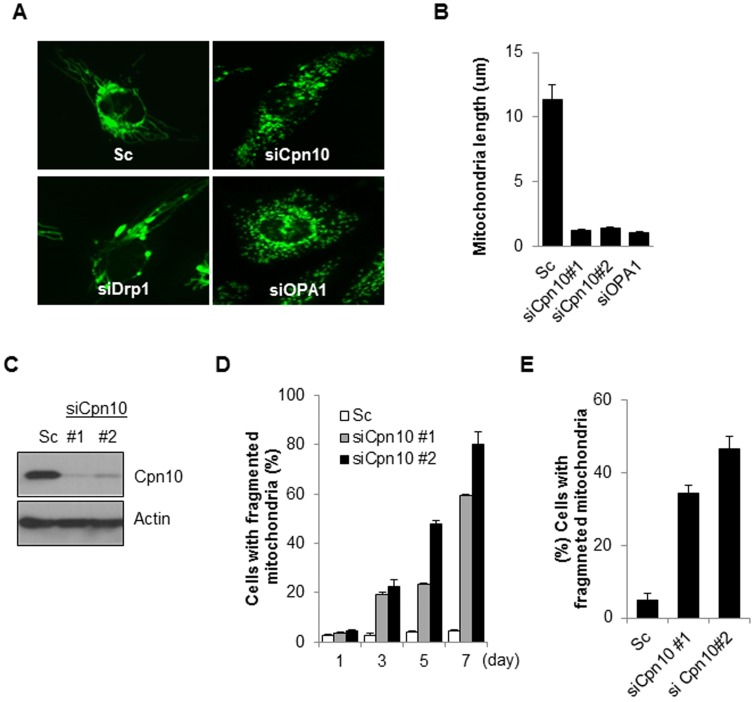
Down-regulation of Cpn10 induces mitochondrial fragmentation in neuroblastoma cells. (A, B) SK-N-MC cells stably expressing mito-YFP (SK/mito-YFP) were transfected with either a control scrambled siRNA (Sc) or a specific siRNA against Cpn10 for 5 days. Then mitochondrial morphology (A) and mitochondrial length (B) were examined with a fluorescence microscope. Both Drp1 siRNA (siDrp1) and Opa1 siRNA (siOpa1) were used as positive controls. (C) The reduced expression of Cpn10 by siRNA was confirmed by Western blotting. (D) SK/mito-YFP cells were transfected with scrambled siRNA or Cpn10 siRNAs (si#1, si#2). And the mitochondrial fragmentation was observed by a fluorescence microscopy at the indicated time points. (E) SH-SY5Y cells were transfected with either a control scrambled siRNA (Sc) or specific siRNAs against Cpn10 (siCPN10 #1, #2). After 5 days, the cells were stained with Mito-tracker (100 nM), and the cells containing fragmented mitochondria were counted using a fluorescence microscope. Data are represented as the mean ± SEM. (n>3).

Inhibition of mitochondrial chaperone proteins, such as DnaJA3 and mortalin induced regulate mitochondrial dynamics [18], [29]. As Cpn10 is a mitochondrial chaperone protein, we examined the effect of a loss of Cpn10 on mitochondrial dynamics. SK-N-MC cells stably expressing mito-tracker fused YFP protein (SK/mito-YFP) were transfected with siRNA against Cpn10 (siCpn10), and their mitochondrial morphology was observed. Depletion of Drp1 resulted in mitochondrial elongation, whereas loss of Opa1 promoted mitochondrial fragmentation as expected (Figure 1A). We also found that mitochondria were massively fragmented following Cpn10 knock down in cells, in a manner as similar to Opa1 knock downs cells (Figure 1A–1D). We further confirmed the Cpn10 knock down effect in other neuroblastoma cells. Consistent with previous results, reduced expression of Cpn10 markedly increased mitochondrial fragmentation in SH-SY5Y cells (Figure 1E). These results suggest that down-regulation of Cpn10 promotes mitochondrial fission in neuroblastoma cells.

### Inhibition of Drp1 suppresses Cpn10 knock down-mediated mitochondria fission and dysfunction

**Figure 2:**
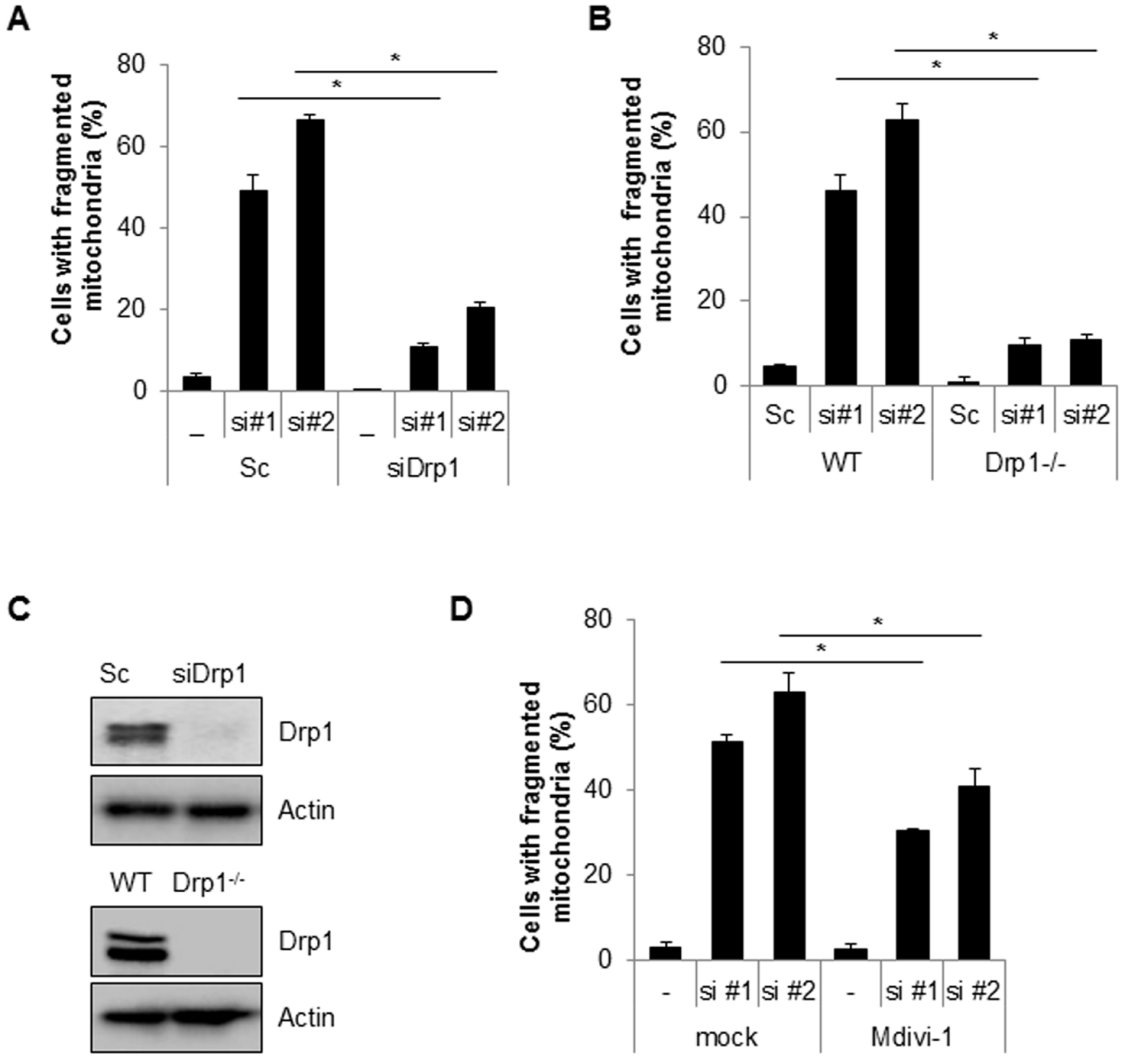
Inhibition of Drp1 suppresses mitochondria fragmentation induced by loss of Cpn10. (A) Drp1 siRNA was co-transfected with either scrambled siRNA (Sc) or Cpn10 siRNA (#1, #2) in SK/mito-YFP cells. 5 days later, the cells with fragmented mitochondria were counted under a fluorescence microscopy. (B) Wild type MEF (WT) and Drp1 deficient MEF (Drp1^-/-^) cells were transfected with either a control scrambled siRNA (Sc) or specific siRNAs against Cpn10 (siCPN10 #1, #2). After 5 days, the cells were labeled with a fluorescence MitoTracker (100 nM) to observe mitochondrial morphology. The cells with fragmented mitochondria were counted under a fluorescence microscopy. (C) The reduced expression of Drp1 in Drp1 siRNA transfected cells and in Drp1 knock out MEF cells was confirmed by Western blotting. (D) SK/mito-YFP cells transfected with scrambled siRNA (Sc) or Cpn10 siRNA (si#1, si#2) were treated with a Drp1 inhibitor, Mdivi-1 (20 µM). The cells with fragmented mitochondria were counted under a fluorescence microscope. Data are represented as the mean ± SEM. (n>3) and were considered significant at a value of *p<0.02.

**Figure 3:**
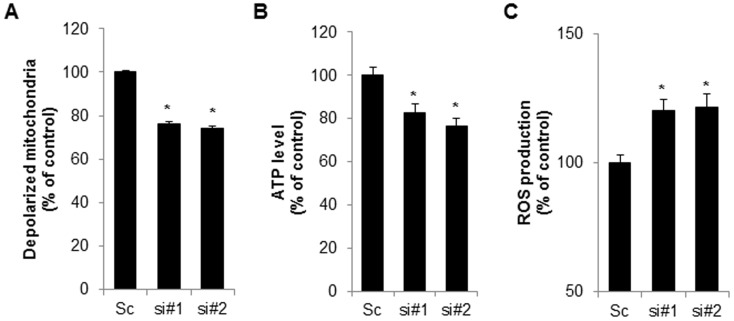
Down-regulation of Cpn10 promotes mitochondrial dysfunction in SK-N-MC cells. (A-C), SK-N-MC cells were transfected with a control scrambled (Sc) or Cpn10 siRNA (si #1, si #2). After 5 days, the alteration of mitochondrial membrane potential was monitored by the MitoProbe JC-1 assay (A). The cellular total ATP level was examined by an ATP bioluminescence detection assay (B). The Intracellular ROS level was measured by a DCFH-DA fluorescence ROS detection assay (C). Data are represented as the mean ± SEM (n>3), and were considered significant at a value of *p<0.05.

Drp1 is a key player in the machinery of mitochondrial fission. To investigate the effects of Drp1 on siCpn10-mediated mitochondrial fission, Cpn10 siRNAs were transfected with or without Drp1 siRNA, into SK/mito-YFP cells. The results showed that a loss of Drp1 expression strongly suppressed Cpn10 knock down-mediated mitochondrial fission (Figure 2A). These effects were further confirmed in Drp1-deficient cells. Either wild type (WT) or Drp1-deficient (Drp1^-/-^) mouse embryonic fibroblast (MEF) cells were transfected with siCpn10, and mitochondrial morphology was monitored. Consistently, siCpn10-induced mitochondrial fragmentation was completely blocked in Drp1^-/-^ MEF cells when compared with control cells (Figure 2B). The inhibition of Drp1 expression in siDrp1 transfected cells and Drp1^-/-^ cells was addressed using Western blot analysis (Figure 2C). Finally, we enhanced the effect of Drp1 with a selective chemical inhibitor of Drp1, Mdivi-1 [30]. Similar with Drp1 down-regulation experiment, Mdivi-1 treatment significantly reduced mitochondria fragmentation in Cpn10 known down cells (Figure 2D). Taken together, these results suggested that Drp1 plays a crucial role in regulating mitochondrial fragmentation in Cpn10 knock down cells. Mitochondrial functions are also influenced by mitochondrial dynamics. Since excessive mitochondrial fragmentation increases mitochondrial dysfunctions, we next examined the effect of Cpn10 knock down on mitochondrial function. Both mitochondrial membrane potential and total cellular ATP level were measured in Cpn10 knockdown cells. As shown in Figure 3, loss of Cpn10 significantly reduced cellular ATP level as well as mitochondrial membrane potential in SK-N-MC cells (Figure 3A, 3B). In addition, depletion of Cpn10 slightly increased ROS production (Figure 3C), suggesting that down-regulation of Cpn10 associated with mitochondrial dysfunctions in neuroblstoma cells.

### Down-regulation of chaperonin10 promotes 3-NP-mediated mitochondrial dysfunction

**Figure 4:**
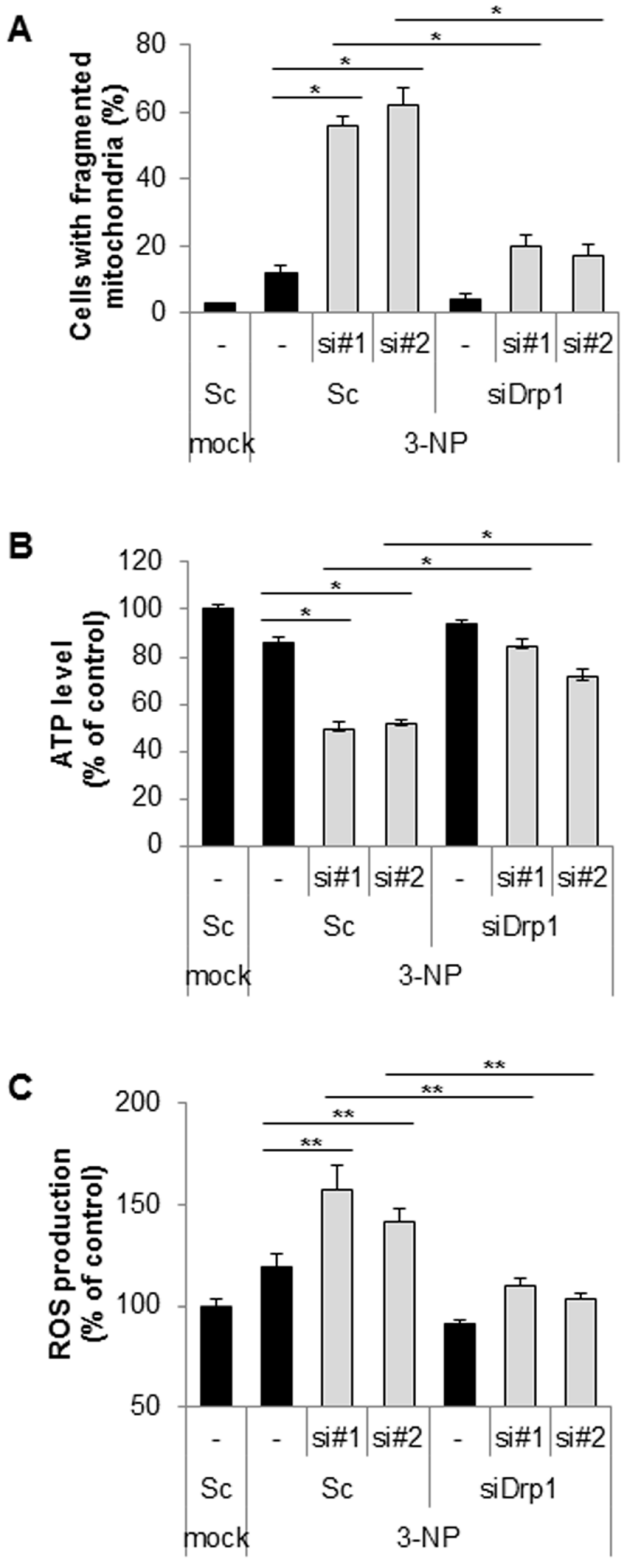
Suppression of Cpn10 exacerbates 3-NP-mediated mitochondrial dysfunction in SK-N-MC cells. (A) SK-N-MC cells were transfected with either scrambled siRNA or Cpn10 siRNA (si#1, si#2) with or without Drp1 siRNA (siDrp1) for 5 days. The cells were further exposed to 3-NP (10 mM) for 8 hr, then cells with fragmented mitochondria were counted with a fluorescence microscope. (B, C) SK-N-MC cells were transfected with either scrambled siRNA or Cpn10 siRNA (si#1, si#2) with or without Drp1 siRNA (siDrp1) for 5 days, then cells were further exposed to 3-NP (10 mM) for 8 hr. The total cellular ATP level was measured by an ATP bioluminescence detection assay (B). The intracellular ROS level was determined by a DCFH-DA fluorescence ROS detection assay (C). Data are represented as the mean ± SEM (n>3), and were considered significant at a value of *p<0.02, **p<0.05.

Chaperone family proteins can prevent the aggregation of polyglutamine (polyQ), which is linked with Huntington's disease (HD)-like pathology and symptoms. 3-nitropropionic acid (3-NP) serves as an experimental model of HD and induces abnormal mitochondria morphology and neurotoxicity [14], [31]. Thus, we further investigated the effect of Cpn10 knock down on 3-NP-mediated mitochondrial dysfunction. As shown in Figure 3A, down-regulation of Cpn10 synergistically enhanced mitochondrial fragmentation in 3-NP treated cells when compared with control cells (Figure 4A). In accordance with the previous results, the Cpn10 knock down–mediated mitochondrial fragmentation was highly suppressed by Drp1 inhibition in 3NP-treated cells (Figure 4A). Interestingly, the reduced ATP level and increased ROS production by siCpn10 were much more impaired by 3-NP treatment (Figure 3B and 3C). However, both ATP reduction and ROS induction were reinstated by inhibition of Drp1 in 3-NP-treated cells (Figure 4B and 4C). Collectively, these results suggest that Cpn10 knock down exacerbates 3-NP-mediated mitochondrial dysfunction in neuroblastoma cells.

### Down-regulation of Cpn10 potentiates 3-NP-mediated neurotoxicity

**Figure 5:**
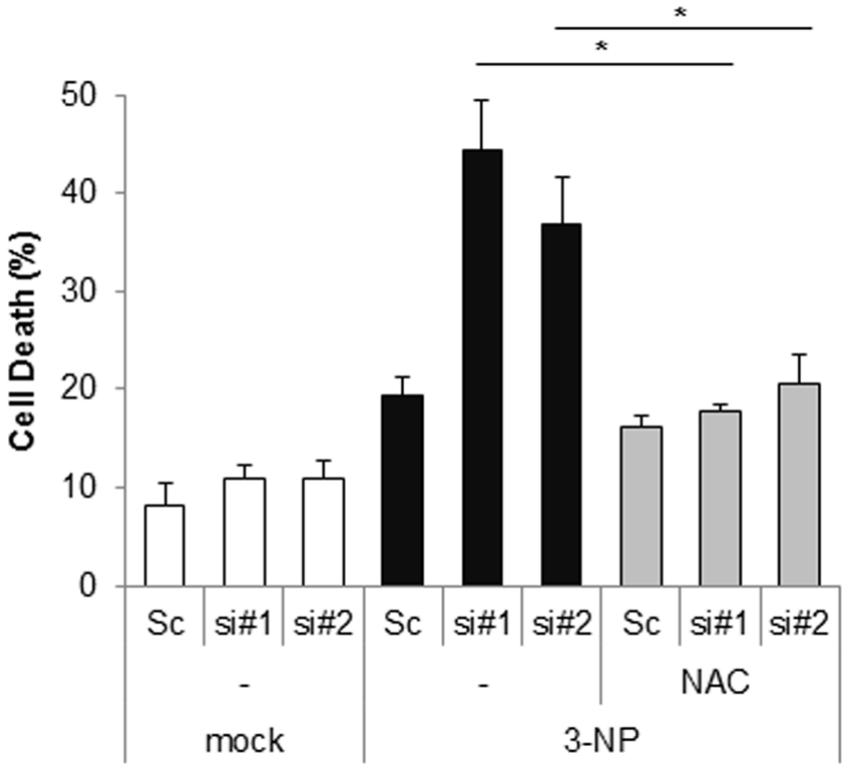
Loss of Cpn10 potentiates 3-NP-mediated cell death in SK-N-MC cells. (A) SK/mito-YFP cells were transfected with scrambled siRNA (Sc) or Cpn10 siRNA (si#1, si#2). After 5 days, the cells were further incubated with 3-NP (10 mM) in the presence or absence of a ROS inhibitor, NAC (1 mM) for 24 hr. Then cells were stained with Annexin V and propidium iodide to measure cell viability by a flow cytometric analysis. Data are represented as the mean ± SEM (n>3), and were considered significant at a value of *p<0.02.

Excessive mitochondrial fragmentation leads to mitochondrial dysfunction and subsequent cell death, therefore, we additionally examined the effect of Cpn10 knock down on 3-NP-induced cell death. SK-N-MC cells transfected with siCpn10 were treated with 3-NP. As shown in Figure 5, depletion of Cpn10 synergistically increased cell death in response to 3-NP in SK-N-MC cells. Interestingly, the cell death was remarkably suppressed by treatment with an ROS scavenger, N-acetylcystein (NAC) (Figure 5). These results suggest that down-regulation of Cpn10 potentiates 3-NP-induced neurotoxicity via excessive ROS production (Figure 5).

## Discussion

Altered mitochondrial dynamics are associated with mitochondrial dysfunctions in neurodegenerative diseases. In this study, we showed that down-regulation of Cpn10 promotes mitochondrial fission and potentiates neurotoxin-induced mitochondrial dysfunction and cell death. Cpn10 plays many roles in mitochondrial homeostasis. Cpn10 is considered a cooperating partner of HSP60 in protein folding processes [32]. The HSP60-Cpn10 protein complex accelerates the folding of polypeptides imported into mitochondria and reduces aggregation of unfolded inactive polypeptides. It has been reported that expression of mitochondrial HSP proteins is up-regulated to protect against cellular damage following global brain ischemia [33]. Overexpression of Cpn10 and HSP60 suppresses cytotoxicity by inhibiting mitochondrial depolarization and modulating mitochondrial Bcl-2 family proteins in cardiomyocytes [34]. Ectopic expression of Cpn10 increases Bcl-xL protein levels and restores the mitochondrial membrane potential as well as reducing caspase activation in doxorubicin-treated cells [24]. In accordance with results, we have found that loss of Cpn10 promotes mitochondrial dysfunction and potentiates cytotoxicity in neuroblastoma cells. Down-regulation of Cpn10 synergistically increased mitochondrial fragmentation and dysfunction following 3-NP or 6-hydroxyl dopamine treatment (Figure 3 and data not shown). Our data presented here further emphasize the importance of Cpn10 in mitochondria. Cpn10 is overexpressed during carcinogenesis of the large bowel and uterine exocervix [35]. In addition, Cpn10 is up-regulated by neuronal vesicular cell trafficking and neuronal synaptic plasticity [36]. Therefore, further studies on the role of Cpn10 in regulating expression in physiology, and patho-physiological conditions will be helpful in understanding its functions and these processes.

Recently our group reported that loss of mitochondrial chaperone proteins could efficiently induce mitochondrial fission and dysfunction in neuronal cells [18]. Despite the known role of Cpn10 in mitochondria, the molecular mechanism underlying mitochondrial fragmentation by Cpn10 knock down is still unknown. Loss of Cpn10 induces mitochondrial fission in a Drp1-dependent manner (Figure 2). Drp1 is a large GTPase protein and several post-translational modifications such as phosphorylation, S-nitrosylation, ubiquitination and O-Glcnacylation of Drp1 protein modulate its GTPase activity [37]–[41]. Among them, phosphorylation is thought to be an important mechanism of Drp1 regulation. The phosphorylation at Serine 616 on Drp1 by many kinases such as CDK1, ERK1/2 and PKC-δ enhances the fission activity of Drp1 in different conditions [42]–[44]. Phosphorylation of Drp1 at another site (Serine 637) by PKA and CamK1-α has shown the opposite effect on Drp1 activation. Phosphorylation of Drp1 by PKA inhibits Drp1 activity, while phosphorylation by CamK1-α increases Drp1 activity [45]–[46]. More recently, Chou et al have reported that GSK3β also mediates the phosphorylation of Drp1 at Serine 693. The phosphorylation of Drp1 by GSK3β inhibits Drp1 function and elongates mitochondria in response to oxidative stress [47]. It has been suggested that Cpn10 could regulate cellular signaling pathways. Cpn10 is found in secretory granules as well as the mitochondrial matrix. Erythropoietin treatment highly promotes the expression and secretion of Cpn10 in endothelial cells [48]. Interestingly, Cpn10 treatment increases phosphorylation of GSK3β and induces cell differentiation [49]. Therefore, the possibility of GSK3β-mediated regulation of mitochondrial dynamics by Cpn10 ought to be elucidated.

In conclusion, we have demonstrated that down-regulation of Cpn10 leads to mitochondrial dysfunctions through mitochondrial fragmentation. In addition, this down-regulation potentiates neurotoxin-mediated neurotoxicity.
